# Fault Diagnosis of Rotating Machinery Using an Optimal Blind Deconvolution Method and Hybrid Invertible Neural Network

**DOI:** 10.3390/s24010256

**Published:** 2024-01-01

**Authors:** Yangde Gao, Zahoor Ahmad, Jong-Myon Kim

**Affiliations:** 1 Department of Electrical, Electronic and Computer Engineering, University of Ulsan, Ulsan 44610, Republic of Korea; gaoyangdephd@gmail.com (Y.G.); zahooruou@mail.ulsan.ac.kr (Z.A.); 2 Prognosis and Diagnostics Technologies Co., Ltd., Ulsan 44610, Republic of Korea

**Keywords:** fault diagnosis, optimal adaptive maximum second-order cyclostationarity blind deconvolution, health index, hybrid invertible neural network

## Abstract

This paper proposes a novel approach to predicting the useful life of rotating machinery and making fault diagnoses using an optimal blind deconvolution and hybrid invertible neural network. First, a new optimal adaptive maximum second-order cyclostationarity blind deconvolution (OACYCBD) is developed for denoising vibration signals obtained from rotating machinery. This technique is obtained from the optimization of traditional adaptive maximum second-order cyclostationarity blind deconvolution (ACYCBD). To optimize the weights of conventional ACYCBD, the proposed method utilizes a probability density function (PDF) of Monte Carlo to assess fault-related incipient changes in the vibration signal. Cross-entropy is used as a convergence criterion for denoising. Because the denoised signal carries information related to the health of the rotating machinery, a novel health index is calculated in the second step using the peak value and square of the arithmetic mean of the signal. The novel health index can change according to the degradation of the health state of the rotating bearing. To predict the remaining useful life of the bearing in the final step, the health index is used as input for a newly developed hybrid invertible neural network (HINN), which combines an invertible neural network and long short-term memory (LSTM) to forecast trends in bearing degradation. The proposed approach outperforms SVM, CNN, and LSTM methods in predicting the remaining useful life of bearings, showcasing RMSE values of 0.799, 0.593, 0.53, and 0.485, respectively, when applied to a real-world industrial bearing dataset.

## 1. Introduction

Due to industrialization, rotating machinery is now widely used in engines, aircraft, and other fields. As rotating machinery works under severe conditions over extended periods, it is vulnerable to failure [1,2,3,4]. Fault diagnosis employs various methods to analyze sensor data, enhancing machinery operation reliability and reducing maintenance costs by minimizing damage. In general, fault diagnosis involves three main stages: signal acquisition, feature extraction, and fault recognition. Sensors are used to monitor rotating machinery, collecting datasets that are processed by software. Signal processing is used to eliminate noise and extract vital signals. Numerous prognostic approaches are available to analyze diverse features derived from degradation data and assess health conditions. Wavelet decomposition can extract effective features in the time–frequency domain that align with machinery degradation patterns. A blind deconvolution algorithm is used to quantify impulsive signatures for early-stage fault diagnosis [5,6,7].

As a commonly used blind deconvolution algorithm, minimum entropy deconvolution (MED) has been widely applied to rotating machinery [8,9]. While MED uses kurtosis maximization to distinguish weak impulse signals from noise, it still faces challenges related to filter length and excessive kurtosis results. To address these issues and enhance performance, maximum correlated kurtosis deconvolution (MCKD), which leverages correlated kurtosis—a parameter more sensitive to vibration signals during specific time intervals—was introduced. Additionally, multipoint optimal minimum entropy deconvolution (MOMEDA) has been proposed, focusing on multipoint kurtosis. These methods excel at extracting impulse signals from mixed signals using an iteratively evaluated index [10,11,12]. From the cyclostationarity view, MCKD does not explicitly investigate its statistical nature, and MOMEDA is based on a periodic criterion to harness the power of cyclostationarity. By comparison, maximum second-order cyclostationarity blind deconvolution (CYCBD) can iteratively resolve an eigenvalue problem [13,14]. As CYCBD can only amplify a fault signature with a specified cyclic frequency, the cyclic frequency needs to be determined in advance, and the ideal CYCBD performance is therefore influenced by prior cyclic frequency. In addition, filter length can affect CYCBD’s denoising capabilities, with slight deviations leading to completely different results. To overcome these drawbacks, adaptive maximum second-order cyclostationarity blind deconvolution (ACYCBD) has been proposed to filter noise, based on an autocorrelation function of a morphological envelope. A cyclic frequency estimation is then applied in the architecture of ACYCBD while a search strategy is deployed to adaptively select the filter length and maintain a balance between performance and time costs when compared with CYCBD [15,16,17].

After filtering processing, a health index was used to assess instant degradation and overall health conditions before applying deep learning to the prediction of the RUL of rotating machinery to enhance safety. This index plays a pivotal role in tracking the machinery’s degradation. An effective health strategy can analyze critical equipment degradation and assess various health conditions. Given the complexity of degradation, designing an efficient health index that accurately represents machinery health states remains challenging and requires incorporating metrics such as the root mean square (RMS) and kurtosis. Subsequently, machine learning or deep learning techniques are used to predict the health conditions of the rotating machinery. This hybrid approach can effectively achieve prognostics and health management (PHM) of rotating machinery, even under varying operating conditions [18,19,20,21,22].

Over the past decade, researchers have developed effective PHM measures to assess the health state of machinery. Support vector machines (SVMs) have successfully estimated the condition of rotating machinery and analyzed convex optimization problems in conjunction with empirical mode decomposition (EMD) [23]. As a special supervised machine learning algorithm, SVM can make use of both margin maximization and support vectors to achieve clear separation in classification and regression tasks. Nevertheless, high computational expenses and challenges in model selection make SVM unsuitable for handling large datasets. In response, an optimal least-squares support vector machine was developed to optimize health indices, identify a hyperplane, and enhance forecasting performance. This approach can be used to create a novel health index using real bearing-failure data [24].

Except for machine learning methods, deep learning methods have been widely applied to prognostics for health monitoring in recent years. As a classic deep learning method, a convolutional neural network (CNN) utilizes the advantages of multiple neural layers to represent input data in feature values and reduce higher numbers of dimensions and improve prognostic recognition [25,26,27,28,29]. However, CNNs suffer from overfitting, exploding gradients, and class imbalances that reduce recognition performance. To improve the quality of a traditional CNN, a multiscale convolutional neural network was designed to estimate the remaining useful life (RUL) of bearings. Such networks effectively and simultaneously integrate global and local information, resulting in enhanced prediction performance [26]. Similar research, utilizing multi-source sensors, has shown that an improved deep CNN can effectively extract data from multiple sensing sources for health-state monitoring. This approach retains the advantages of multi-source data while overcoming issues related to overfitting due to spatial fluctuations, ultimately leading to efficient and accurate health monitoring [27]. While these methods can achieve high prediction accuracy for estimates of the RUL of rotating machinery, they often require their performance to be maintained under numerous assumptions and with large datasets. To address data-related challenges, a transferable neural network method was used to predict the RUL of rotating machinery. This approach minimizes divergence in distance and conditional probability distributions to assess the health index, reducing reliance on the availability of high-quality data. The results demonstrate its effectiveness and superiority compared with existing methods [30,31,32].

Long short-term memory (LSTM) networks can use their architectural strengths to retain long-term memories of bearing conditions. LSTM networks effectively address the limitations and instability issues associated with predicting the RUL of rolling bearings, producing superior forecasting performance [33,34,35,36]. The LSTM method demands greater memory to handle time sequences. Bearing degradation, which is a measure of cumulative damage, implies that degradation in the next step is influenced by previous damage. This requires the ability to infer input features from given outcome values, resulting in mutual interference between input features and outcome values. To address this challenge, an invertible neural network (INN) was designed to induce invertible transformation, which enables a mutual exchange between input features and outcome values, effectively harnessing the advantages of correlation information to enhance recognition performance. [37,38,39]. Bayesian optimization was used to enhance the performance of the INN. These concepts and strategies have proved to be highly beneficial [40,41].

In this paper, ACYCBD has been successfully applied to the detection of vibration-bearing faults characterized by cyclic frequencies. It can identify fault features using the envelope harmonic product spectrum, even without prior information. However, as machinery can experience wear and tear under pressure and high-speed conditions, bearing degradation can result in fault features at varying cyclic frequencies [15,16]. To enhance and optimize the adaptability of the ACYCBD method for such scenarios, we employed a Monte Carlo probability density function (PDF) [42]. We chose cross-entropy [43] to replace the conventional iteration process in ACYCBD as this modification optimizes filter coefficients and enhances the filtering performance of vibration signals across a wide range of frequencies. This approach further advances the application of ACYCBD in detecting bearing degradation. We proposed optimal adaptive maximum second-order cyclostationary blind deconvolution (OACYCBD) to effectively filter noise from signals associated with bearing degradation. OACYCBD proved particularly well-suited for analyzing denoised signals stemming from bearing degradation across a wide range of frequencies [44,45,46].

After successfully filtering out health-sensitive signals resulting from the degradation of bearings, a health index was applied to promptly assess the state of degradation and the overall health condition. The index plays a pivotal role in assessing degradation. It incorporates peak properties and the square of the arithmetic means to process denoised signals and analyze the health condition of the bearing. It provides a time-domain measure of bearing degradation, considering factors such as pressure and speed, allowing it to depict various health condition stages under different circumstances. It also exhibits greater sensitivity to bearing degradation.

To enhance PHM capabilities for bearing degradation, this study was designed to enhance bearing degradation prediction by integrating an INN with LSTM, resulting in a hybrid invertible neural network (HINN) that facilitates the mutual exchange of input features and outcome values and can seamlessly infer input features from given outcome values. This aligns with the damage-accumulation process observed in bearing degradation. By leveraging an invertible transformation, the HINN was designed to optimize forecasting performance.

In brief, this paper makes the following major contributions:

1. Although ACYCBD excels at identifying fault features in vibration signals with cyclic frequencies, bearing degradation involves the accumulation of fault features at variable cyclic frequencies, particularly under high pressures and speeds. To enhance ACYCBD’s filtering performance in this context, we introduced an OACYCBD method. In OACYCBD, we used a probability density function (PDF) of Monte Carlo to assess condition characteristics and detect subtle dissimilarities in vibration signals. We also replaced the traditional iteration process of ACYCBD with cross-entropy to optimize filter coefficients. These measures collectively led to a significant improvement in OACYCBD’s ability to extract features from bearing degradation, surpassing the performance of ACYCBD, particularly when dealing with variable frequencies.

2. Once the noise signals stemming from bearing degradation have been filtered, the next step involves identifying peaks within the denoised signals and taking advantage of their peak properties. These peaks are then combined with the square of the arithmetic mean to produce a novel health index. A robust index is instrumental in analyzing critical aspects of equipment degradation and quantifying various stages of health conditions. We designed an index that evaluates bearing degradation across different pressure and speed scenarios in the time domain, thereby providing valuable insights into health conditions under diverse conditions.

3. To enhance PHM capabilities for bearing degradation, we leveraged the strengths of both an INN and LSTM to create a HINN. The HINN architecture allows for the mutual exchange of input features and outcome values. This unique characteristic gives the HINN the ability to effectively model the accumulation of damage during bearing degradation and outperform the LSTM method in terms of predictive performance.

The remainder of the paper is organized as follows. In Section 2, the OACYCBD filtering method is derived from ACYCBD. After filter processing, a novel health index is designed to assess bearing degradation in Section 3. Section 4 describes the novel HINN forecasting model of bearing degradation. Experimental results and discussions are presented in Section 5. Finally, conclusions and avenues for future research are explored in Section 6.

## 2. Optimal Adaptive Maximum Second-Order Cyclostationarity Blind Deconvolution

ACYCBD has been successfully applied to the detection of vibration-bearing faults characterized by cyclic frequencies. However, as machinery can experience wear and tear under pressure and high-speed conditions, bearing degradation can result in fault features at varying cyclic frequencies. To enhance and optimize the adaptability of the ACYCBD method for such scenarios, we proposed optimal adaptive maximum second-order cyclostationary blind deconvolution (OACYCBD) to effectively filter noise from signals associated with bearing degradation. OACYCBD proved particularly well-suited for analyzing denoised signals stemming from bearing degradation across a wide range of frequencies.

### 2.1. Review of ACYCBD

In the ACYCBD method, an envelope harmonic product spectrum (EHPS) was used to extract hidden cyclic frequencies from vibration signals when compared with the original CYCBD method. ACYCBD is a modified version of CYCBD in blind deconvolution theory [15,16,17]. The basic theory of blind deconvolution is to obtain a source signal $s_{0}$, from an observation signal x. The process can be expressed mathematically as:

(1) $$s=x*h=\left(s_{0}*g\right)*h\approx s_{0}$$

where s is the estimated input, g is the unknown impulse response, h is the inverse finite impulse response filter, and ∗ is the convolution operation. The convolution operation for matrix form can be expressed as:

(2) $$\left\{\begin{array}{c} s\left[L-1\right]\\ \vdots \\ s\left[N-1\right] \end{array}\right\}=\left[\begin{array}{ccc} x\left[L-1\right] & \cdots & x\left[0\right]\\ \vdots & \ddots & \vdots \\ x\left[N-1\right] & \cdots & x\left[N-L-2\right] \end{array}\right]\left\{\begin{array}{c} h\left[0\right]\\ \vdots \\ h\left[L-1\right] \end{array}\right\}$$

where N is the length of x and L is the length of h. An appropriate criterion is necessary to obtain the optimal inverse filter, $h_{0}$, and the optimal function is shown in Equation (3):

(3) $$h_{0}=\underset{h}{\mathrm{arg max}}\left[O(h)\right]$$

where $O\left(h\right)$ is the objective function for the performance of blind deconvolution. The ACYCBD is derived from CYCBD, and the important concept of cyclic frequency is defined as:

(4) $$\alpha =\frac{1}{T_{s}}$$

where $T_{s}$ is the period of fault impact. Second-order cyclostationarity ($ICS_{2}$) is defined as:

(5) $${ICS}_{2}=\frac{\sum_{k>0}{\left|c_{s}^{k}\right|}^{2}}{{\left|c_{s}^{0}\right|}^{2}}$$

with

(6) $$c_{s}^{k}=\langle {\left|s\right|}^{2},e^{j2\pi k\alpha n}\rangle$$

(7) $$c_{s}^{0}=\frac{{\left\|s\right\|}^{2}}{N-L+1}$$

where ${ICS}_{2}$ is the objective function of deconvolution and the criterion of CYCBD is shown by:

(8) $$h_{0}=\underset{h}{\mathrm{arg max}}{ICS}_{2}$$

The optimal filter coefficient of CYCBD can be obtained from the eigenvalue algorithm (EVA) by addressing the generalized eigenvalue problem using Equations (9) and (10):

(9) $$c_{s}^{k}=\frac{EDs}{N-L+1}$$

(10) $$c_{s}^{0}=\frac{s^{H}s}{N-L+1}$$

with

(11) $$D=diag\left(s\right)=\left[\begin{array}{ccc} \ddots & & 0\\ & s[l] & \\ 0 & & \ddots \end{array}\right]$$

(12) $$E=[\begin{array}{ccc} e_{1}\cdots & e_{k}\cdots & e_{K} \end{array}]$$

(13) $$e_{k}={\left[\begin{array}{cc} e^{-j2\pi k\alpha (L-1)}\cdots & e^{-j2\pi k\alpha (N-1)} \end{array}\right]}^{T}$$

${ICS}_{2}$ can then be expressed as:

(14) $${ICS}_{2}=\frac{s^{H}D^{H}EE^{H}Ds}{{\left|s^{H}s\right|}^{2}}$$

and the final expression calculated by:

(15) $${ICS}_{2}=\frac{h^{H}X^{H}WXh}{h^{H}X^{H}Xh}=\frac{h^{H}R_{XWX}h}{h^{H}R_{XX}h}$$

where $R_{XWX}$ is the weighted correlation matrix and $R_{XX}$ is the correlation matrix, with a weight matrix, W, expressed as:

(16) $$W=\frac{D^{H}{EE}^{H}D}{s^{H}s}$$

The optimal inverse filter $h_{0}$ is equivalent to h from maximizing ${ICS}_{2}$ by resolving the maximum eigenvalue λ.

(17) $$R_{XWX}h=R_{XX}h\lambda$$

The above descriptions constitute CYCBD theory. ACYCBD is a derived version of CYCBD. In ACYCBD, an EHPS is used to extract hidden cyclic frequencies from vibration signals when compared with the original CYCBD method. ACYCBD procedures are described as follows:

Step 1: Set initial parameters for vibration signals x, the convergence criterion $\varepsilon_{0}$, the maximum iteration number $N_{max}$, the filter length L, and the initial filter coefficient h.

Step 2: Calculate a temporary denoising signal *S* from vibration signals by initial parameters.

Step 3: Use the EHPS to estimate the cyclic frequency of denoising signals and analyze the amplitude envelope spectrum.

Step 4: Detect the estimated cyclic frequency with the global maximum amplitude from a specified frequency range in EHPS.

Step 5: Obtain the correlation matrix $R_{XX}$, the weight matrix W, and the weighted correlation matrix $R_{XWX}$, and then update the filter coefficient in the maximum eigenvalue for the eigenvalue problem.

Step 6: Return to Step 2, update the filter coefficient h for the next similar cyclic steps, and end with the convergence criterion.

Step 7: Finish with the output filtered signal.

### 2.2. The Proposed OACYCBD Method

Although ACYCBD can detect bearing faults characterized by cyclic frequencies, challenges arise when dealing with high pressures and speeds. Bearing degradation results in the accumulation of fault features at varying cyclic frequencies, necessitating adaptability enhancements for the ACYCBD method.

To advance the application of ACYCBD in bearing degradation, we introduced OACYCBD, which leverages a Monte Carlo PDF to optimize the filtering process. A probability density function (PDF) of Monte Carlo assesses condition characteristics and detects subtle dissimilarities within vibration signals, optimizing the weight coefficients of ACYCBD.

We also replaced the traditional iteration process of ACYCBD with cross-entropy, which efficiently updated the optimal filter coefficients, leading to improved filtering performance. As a result, OACYCBD excelled at filtering noise signals associated with bearing degradation, particularly when dealing with variable frequency characteristics, and outperformed standard ACYCBD when extracting features from bearing degradation.

Vibration signals refer to those obtained during the deterioration or performance loss of the bearing. A PDF [42] is a mathematical tool to describe the probability distribution of a random variable. In the context of bearing conditions, a PDF can also be used to represent the likelihood of various levels of bearing states under different operating conditions. A PDF therefore proves invaluable in assessing condition characteristics for vibrating bearings.

**Figure 1:**
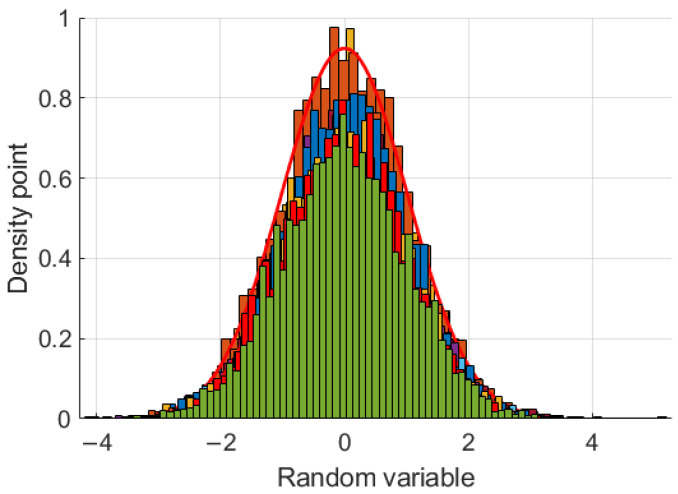
The density distribution of a Monte Carlo simulation using a random variable.

Monte Carlo methods can help quantify subtle differences within the PDFs of vibration signals. Through these methods, a PDF can effectively use the following functions to demonstrate its performance [17].

(18) $$p=(1-e^{-x^{2}/2\delta^{2}})$$

where x is the variable data and δ is the standard deviation. As shown in Figure 1, a PDF can model the density point and slight dissimilarities in a Monte Carlo simulation with a random variable. It can show that δ is critical to performance and requires an updating process in different conditions.

In the context of MED, CYCBD, and ACYCBD filtering methods, the convergence criteria continue to rely on traditional criteria, typically when the satisfaction of certain key values and predefined iteration numbers are involved. However, in recent years, the growing popularity of deep learning has introduced novel approaches to optimization. The use of cross-entropy has gained significant traction within the deep learning community due to its ability to effectively update parameters and yield favorable results [43], such as:

(19) $$loss=TlnY+\left(1-T\right)ln\left(1-Y\right)$$

where T is the targeted value and Y is the prediction value.

**Figure 2:**
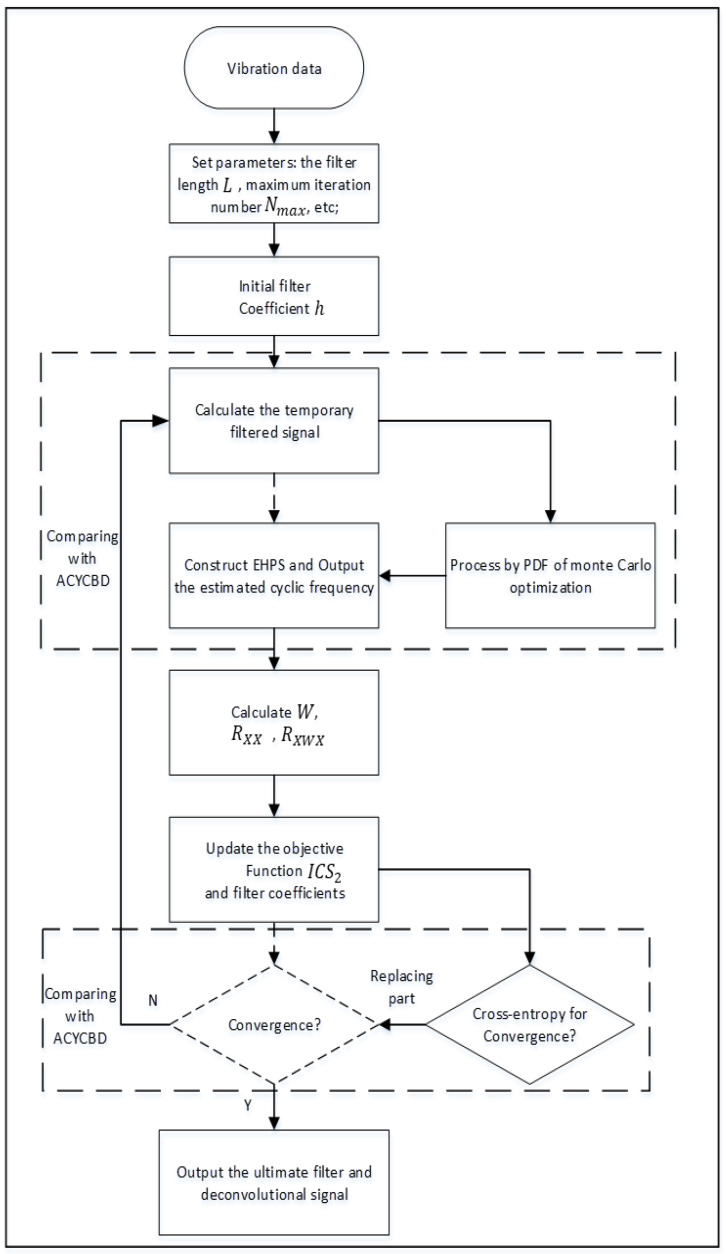
Flowchart of the proposed OACYCBD.

The OACYCBD procedure is shown in Figure 2 and described as follows:

Step 1: Set initial parameters for the vibration signal x, including a convergence criterion $\varepsilon_{0}$, maximum iteration number $N_{max}$, filter length L, and initial filter coefficient h.

Step 2: Calculate the temporary filtering signal *S* from the vibration signals using initial parameters, and then apply a Monte Carlo PDF that optimizes the temporary filtering signals to make an assessment. This can assess condition characteristics and measure slight dissimilarities to estimate signals.

Step 3: Apply an EHPS to estimate the cyclic frequency of estimated signals and analyze the amplitude envelope spectrum.

Step 4: Detect the estimated cyclic frequency with the global maximum amplitude from a specified frequency range in EHPS.

Step 5: Obtain the correlation matrix $R_{XX}$, the weight matrix W, and the weighted correlation matrix $R_{XWX}$, and then update the filter coefficients in the maximum eigenvalue for the eigenvalue problem.

Step 6: Replace the traditional convergence criterion of ACYCBD with cross-entropy, return to Step 2, update the filter coefficient h to the next similar cyclic steps, and end with the convergence criterion.

Step 7: Finish with the output filtered signal.

**Figure 3:**
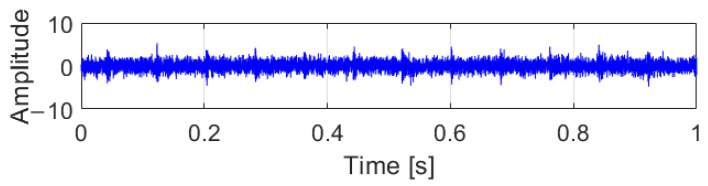
Raw vibration signals.

**Figure 4:**
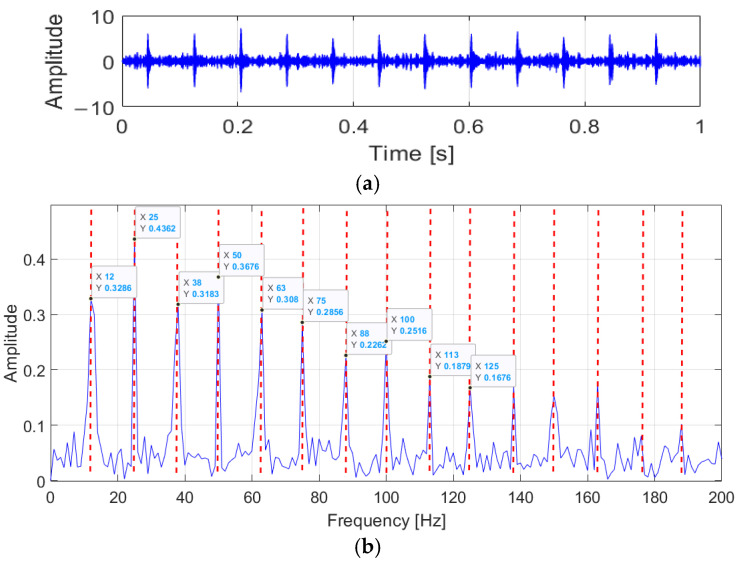
Results from ACYCBD: (**a**) denoised signal in the time domain; (**b**) envelope spectrum.

**Figure 5:**
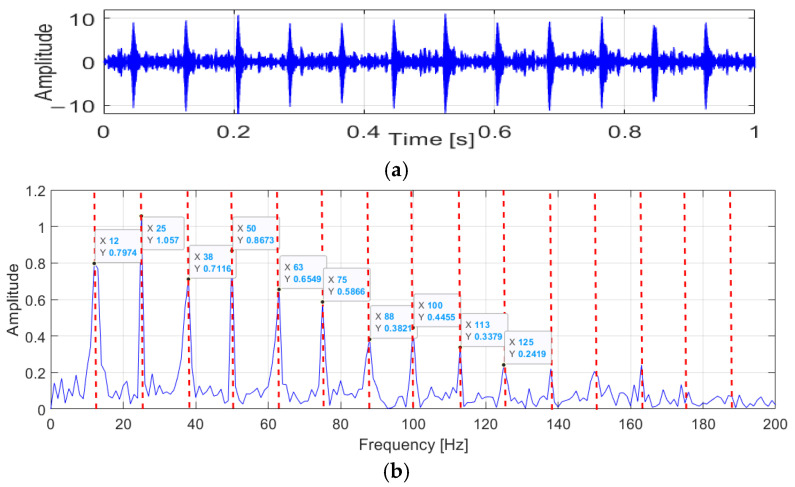
Results obtained from OACYCBD: (**a**) the denoised signal; (**b**) envelope spectrum.

To demonstrate the performance of the proposed methods on filtering noise signals, vibration signals were utilized to verify the effectiveness of OACYCBD and ACYCBD (Figure 3, Figure 4 and Figure 5). In the vibration signals, feature impulse signals were generated when the rollers reached the damage point, but because they were mixed with noise signals, the amplitude of the noise was greater than the pulse signal, and it was not easy to identify the fault information before filtering processing.

The filtering results obtained using ACYCBD are depicted in Figure 4. To further analyze the processed signals, an envelope spectrum was used to derive the autocorrelation function, which effectively revealed energy modulation patterns. In this analysis, the fault frequency had a clear and unmistakable peak at 12 Hz. By calculating frequency multiplications within the interval [12, 25, 38, 50, 63, 75, 88, 100, 113, 125 …] Hz, distinct peaks emerged at frequencies such as 25, 38, 50, …, which were close to double, triple, and quadruple the fault frequency. These observed fault characteristics provided valuable insights for diagnosis and assessment.

Subsequently, the OACYCBD filtering method was used to eliminate noise, and its effectiveness was verified through envelope spectrum analysis. The results were striking, with clear and distinct spectral lines. The fault frequency at 12 Hz was readily identifiable and more pronounced compared with ACYCBD processing. The extraction of frequency multiplications, such as [12, 25, 38, 50, 63, 75, 88, 100, 113, 125 …], was notably accurate, with peak frequencies closely aligned with 1–10 times the fault frequency.

At a peak frequency of 12 Hz, the amplitude achieved by OACYCBD was 0.79, while ACYCBD yielded an amplitude of 0.328. Similar comparisons across various frequencies consistently showed higher amplitudes with OACYCBD. These results unequivocally demonstrated that OACYBD filtering outperformed ACYCBD.

In line with the earlier descriptions, the vibration signals were primarily decomposed into feature responses. Analysis of amplitudes revealed that periodic fault impulses were successfully extracted from noisy signals using both OACYCBD and ACYCBD. However, the envelope spectrum highlights that variable cyclic frequencies are more prominently discernible in OACYCBD. These outcomes firmly establish OACYCBD’s superior performance over ACYCBD, making it better suited for health analysis.

## 3. The Proposed Health Index for Rotating Machinery

After the noise signals from bearing degradation are effectively filtered, deep learning methods can be applied to predictions of bearing degradation. A crucial element in this process is the health index, which is a fundamental component of machinery degradation assessments. An effective health index strategy is pivotal in analyzing critical aspects of equipment degradation and quantifying various stages of health conditions.

Following the filtration of noise signals, a novel health index was introduced to assess the RUL of the machinery. This innovative method operates in the time domain, allowing it to gauge bearing degradation under varying conditions of pressure and speed and reveal the stages of health conditions in different scenarios.

**Figure 6:**
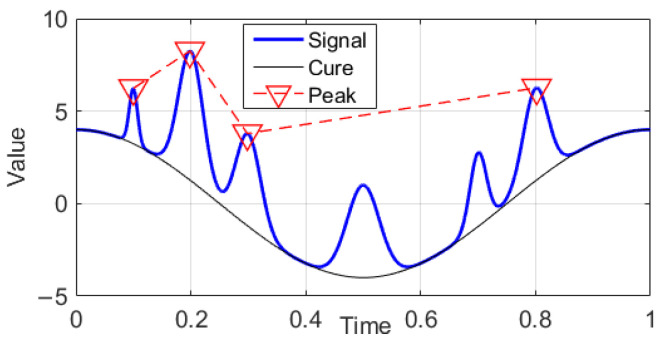
The peak signal theory.

In this paper, a health index was developed based on the “peaks theory”, in which peaks are identified by analyzing changes in the slope or curvature of the signal. Once these peaks are detected, further analysis can be conducted on the identified points [47]. The peak information is shown in Figure 6.

Vibration signals $\left(\begin{array}{ccc} x_{1}, & \cdots , & x_{n} \end{array}\right)$ are treated as input data. First, a search for local maxima (peaks, p) from the input data is conducted, after which the average of peaks is calculated, the variance with input signals is evaluated, and the squares of the variance are summed. Finally, we divide the sum and take the square as the result, as presented in Equation (20).

(20) $$HI=\sqrt{\frac{1}{n}\sum {\left(\frac{1}{p}\sum x_{p}-x\right)}^{2}}$$

## 4. The Hybrid Invertible Neural Network

In this paper, we introduce a HINN designed to address inverse prediction problems in forecasting. Our proposed method incorporates an invertible sub-network capable of performing one-to-one mapping from feature information to an intermediate encoded feature. The model’s scalability is achieved through a combination of the encoded feature’s mixture model and LSTM. Furthermore, invertible flow mapping is facilitated by leveraging theories related to optimal transport and diffusion processes.

**Figure 7:**
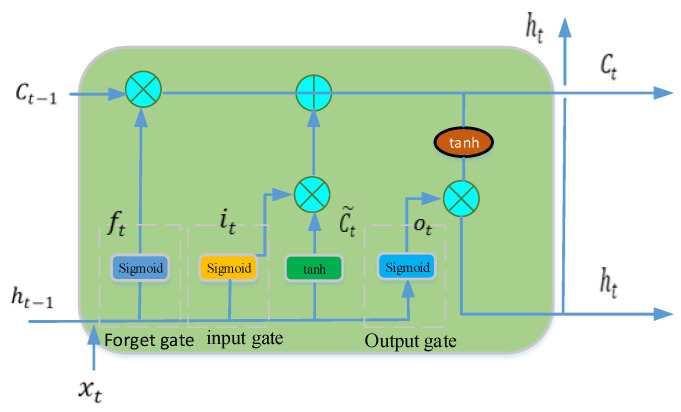
The architecture of LSTM.

LSTM, a specialized model derived from recurrent neural networks (RNNs), has a fundamental structure illustrated in Figure 7. It consists primarily of a cell and three gates: an input gate, an output gate, and a forget gate. These components work together to facilitate the flow of the model’s memory and information [33,34]. The cell within LSTM retains values over arbitrary time intervals, and the three gates play crucial roles in governing the information flow within the cell. Forget gates determine which information to discard from the previous state, input gates determine which pertinent information to incorporate into the current state, and output gates control the crucial information to be included in the current state. This architecture mitigates the limitations of RNNs and enables the retention of essential long-term dependencies for prediction. Detailed mathematical formulations are presented below.

(21) $$f_{t}=sigmoid(W_{f}x_{t}+U_{f}h_{t-1}+b_{f})$$

(22) $$i_{t}=sigmoid(W_{i}x_{t}+U_{i}h_{t-1}+b_{i})$$

(23) $$o_{t}=sigmoid(W_{o}x_{t}+U_{o}h_{t-1}+b_{o})$$

(24) $$\tilde{C_{t}}=tanh(W_{c}x_{t}+U_{c}h_{t-1}+b_{c})$$

(25) $$C_{t}=f_{t}⨀C_{t-1}+i_{t}⨀\tilde{C_{t}}$$

(26) $$h_{t}=o_{t}⨀tanh(C_{t})$$

where $x_{t}$ is the input vector, $f_{t}$ is the forget gate’s activation, $i_{t}$ is the input gate’s activation, $o_{t}$ is the output gate’s activation, $h_{t}$ is a hidden state vector, $\tilde{C_{t}}$ is cell input activation, $C_{t}$ is the cell state vector, and W,U,and b are weight matrices and bias parameters.

**Figure 8:**
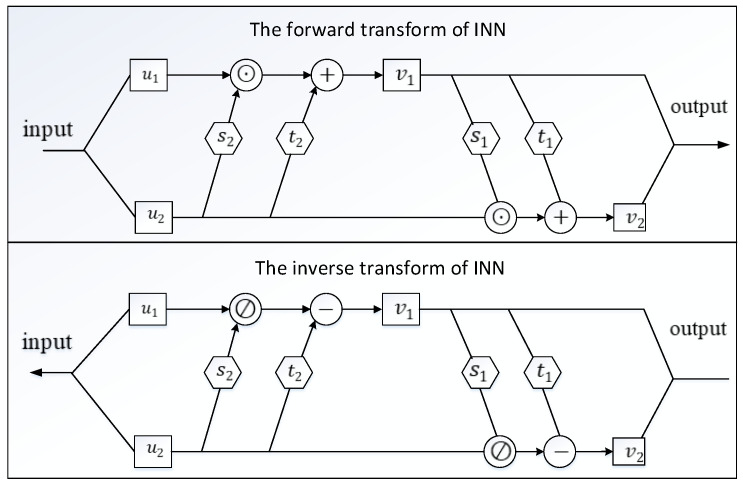
An invertible neural network.

In the basic invertible nonlinear transformation, the forward neural network F(u)=υ, the invertible mapping $F^{-1}(\upsilon )=u$, the probability satisfies $\theta \sim p\left(\theta |x\right)$ and a Gaussian latent variable z, and $\theta =F^{-1}(z;x)$ with $z\sim N\left(z|0,I\right)$ following $\theta \sim p\left(\theta |x\right)$ [48]. $N\left(z|0,I\right)\propto exp({\left\|-(1/2)z\right\|}_{2}^{2}$), the basic building block of the invertible neural network, is the affine coupling layer. The approach works by splitting the input data u into an average of two parts $\left[{u_{1},u}_{2}\right]$, as shown in Figure 8. Using transformation through $s_{i}$, $t_{i}$, the following operation can be described:

(27) $$v_{1}=u_{1}⊙exp(s_{1}(u_{2}))+t_{1}(u_{2})$$

(28) $$v_{2}=u_{2}⊙exp(s_{2}(u_{1}))+t_{2}(u_{1})$$

where ⊙ is element-wise multiplication, the output is $\nu =\left[{v_{1},v}_{2}\right]$, and $F({u_{1},u}_{2})=({v_{1},v}_{2})$.

Rearranging the previous equations, we can recover $\left[{u_{1},u}_{2}\right]$ from $\left[{v_{1},v}_{2}\right]$ to compute the inverse, and the invertible operation can be calculated by:

(29) $$u_{2}={(v}_{2}-t_{1}(v_{1}))⊙exp(-s_{1}(v_{1}))$$

(30) $$u_{1}={(v}_{1}-t_{2}(u_{2}))⊙exp(-s_{2}(u_{2}))$$

(31) $$F^{-1}(({v_{1},v}_{2}))=({u_{1},u}_{2})$$

**Figure 9:**
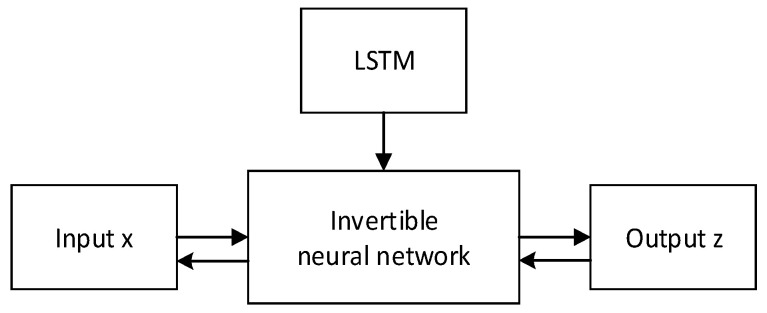
An illustration of the proposed hybrid invertible neural network.

In the HINN architecture shown in Figure 9, we optimized the parameters of the LSTM neural network jointly with those of the INN chain via backpropagation. To incorporate the input data, the proposed method can be augmented by taking $h_{t}$ as an additional input and then calculating the output as

(32) $$v_{1}=u_{1}⊙exp(s_{1}(u_{2},h_{t}))+t_{1}(u_{2},h_{t})$$

(33) $$v_{2}=u_{2}⊙exp(s_{2}(u_{1},h_{t}))+t_{2}(u_{1},h_{t})$$

The entire invertible chain can be expressed as $F(\theta ;h_{t})=z$, together with the inverse operation $F^{-1}(z;h_{t})=\theta$, $\theta \sim p\left(\theta |x\right)$. $N\left(z|0,I\right)\propto exp({\left\|-(1/2)z\right\|}_{2}^{2}$).

## 5. Experimental Validation

**Figure 10:**
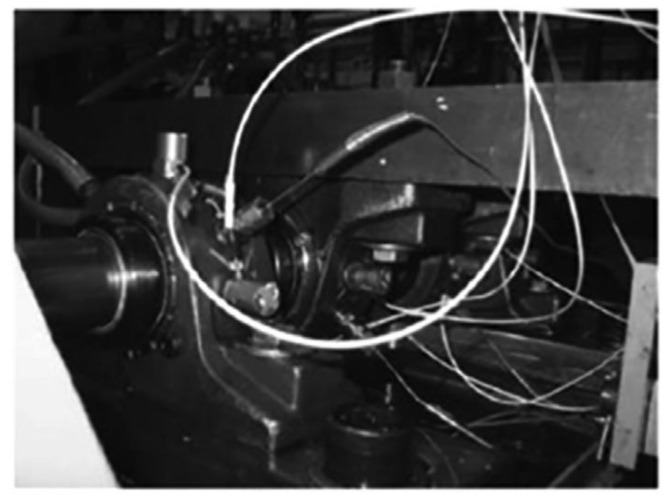
Bearing platform on bearing degradation.

To validate the performance of the proposed prognostic approach, data from the Intelligent Maintenance System (IMS) center at the University of Cincinnati were used [7,16]. The experiment involved four Rexnord ZA-2115 double-row bearings. An AC motor operated at a constant speed of 2000 rpm, and the data were sampled at 20,000 Hz. The applied load was 6000 foot-pounds, and vibration data were monitored using PCB 353B33 High Sensitivity Quartz ICP accelerometers. The experimental platform in Figure 10 provides a basis for comparing the performance of various methods in the context of bearing degradation analysis.

The proposed prognostic approach was validated using IMS run-to-failure data. As discussed above, this approach effectively addressed three critical challenges in the health assessment of bearing degradation:

1. OACYCBD filtering was used to eliminate noise signals from mixed vibration signals characterized by different cyclic frequencies, achieving superior feature extraction.

2. A novel health index was introduced to capture the evolving trend of bearing degradation efficiency.

3. A HINN model was used to predict future degradation trends in the health index for estimating the RUL.

**Figure 11:**
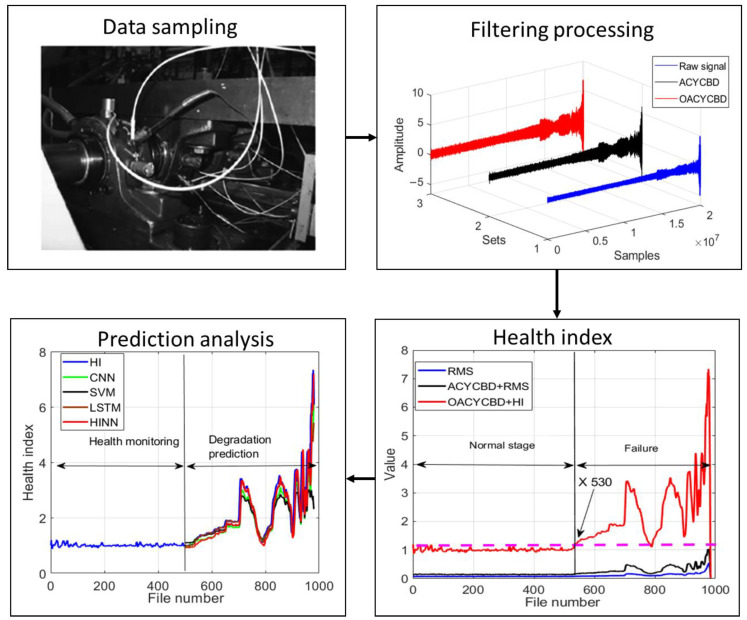
The proposed prognosis framework.

This comprehensive process ensures the accurate assessment of the health condition of vibrating bearings, as depicted in Figure 11.

The run-to-failure experiment can be described using three datasets, with each dataset comprising 984 vibration signal samples. Each 1 s vibration signal sample contained 20,480 data points, and all samples underwent processing using filtering methods.

**Figure 12:**
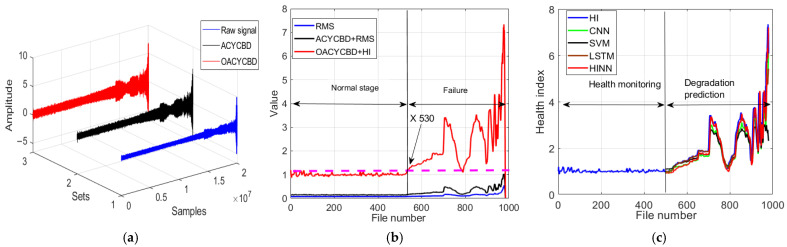
Bearing degradation processing results using different methods: (**a**) filtered signal by OACYCBD, (**b**) health index trend, and (**c**) forecast model using a hybrid INN model.

Figure 12a illustrates the use of a Monte Carlo PDF to optimize the filtering signal during the filtering process, which employed OACYCBD to assess the data. Additionally, cross-entropy was employed to replace the traditional iteration process found in ACYCBD. This effectively filtered noise signals originating from bearing degradation, particularly those with variable frequency characteristics.

After successfully filtering noise from bearing degradation, we proceeded to design a novel health index tailored to assess the RUL. Each sample containing 20,480 data points was transformed into a health index using both the proposed index and RMS.

As illustrated in Figure 12b, the novel index method was capable of quantifying bearing degradation under various pressures and speeds in the time domain. This allowed for the visualization of distinct health condition stages in different scenarios. These conditions include the normal stage and the failure stage. An initial trend in bearing degradation became apparent as early as sample number 530, at which point both OACYCBD and the proposed health index met.

In contrast, when using ACYCBD+RMS and RMS alone, this initial bearing degradation trend was observed to begin with sample number 700. In addition, monotonicity was used to assess the health index construction [49]. The monotonicities were 0.11, 0.16, and 0.24 for RMS, ACYCBD+RMS, and OACYCBD+health index, respectively. The proposed method resulted in higher monotonicity compared with the reference methods. The above description shows that the proposed method is more sensitive to bearing degradation compared with either ACYCBD or RMS.

Finally, the HINN addressed the challenge of inverse prediction in memory-related tasks by framing it as a conditional memory task. To illustrate the prediction process more effectively, we used 980 samples for both training and testing chosen randomly at the ratio of 1:1. The training began with the first sample’s data, which included 20,480 data points, and the testing used the data from the second sample. This process was repeated sequentially until the final sample’s data were used. Each internal segment consists of one sample’s data, totaling 20,480 points.

**Figure 13:**
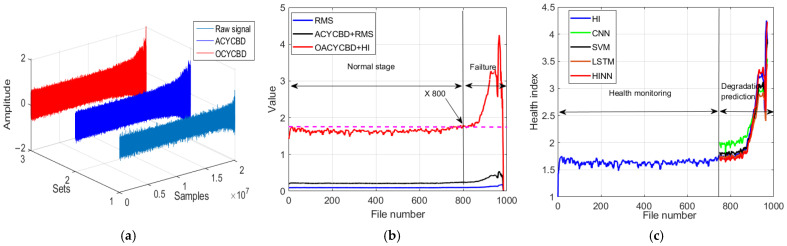
The processing for different methods on bearing degradation: (**a**) denoised signal by OACYCBD, (**b**) health index trend of bearing degradation, and (**c**) forecast model using a hybrid INN model.

**Figure 14:**
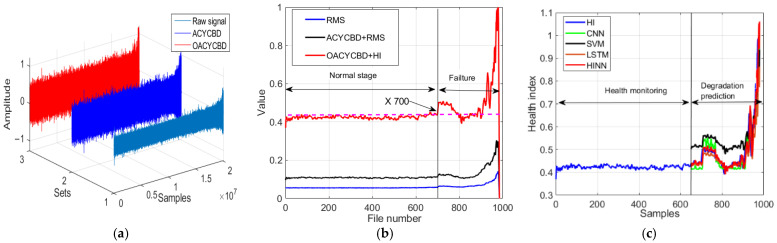
Processing for different methods for bearing degradation: (a) filtered signal by OACYCBD, (**b**) health index trend of bearing degradation, and (**c**) a forecast model on hybrid INN model.

**Figure 15:**
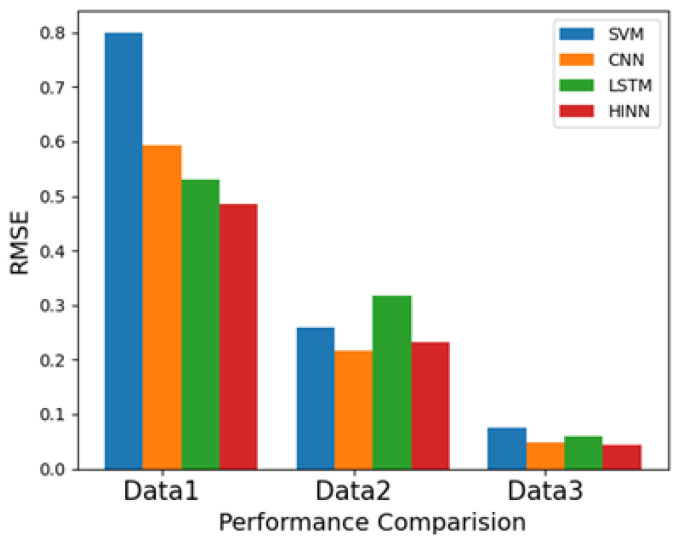
Performance comparison of model prediction.

The model’s ability to handle larger memory neural networks was achieved through a mixture of encoded features, particularly for bearing degradation, as depicted in Figure 12c. The evaluation included data from sample numbers 500 to 980 for degradation prediction. The results demonstrate superior forecasting performance when compared with SVM, CNN, and LSTM methods. The root mean square error (RMSE) evaluates the performance of different methods for forecasting [50,51]. The RMSE was 0.799 for SVM, 0.593 for CNN, 0.53 for LSTM, and 0.485 for HINN in Table 1. This shows that HINN has superior forecasting abilities compared with SVM, CNN, and LSTM methods. We also employed two more datasets to further validate the proposed methods in the context of filtering, health index assessment, and forecasting analysis, as depicted in Figure 13 and Figure 14. RMSE values are summarized in Figure 15 and Table 1.

In Figure 13a, the samples underwent processing through filtering methods. Notably, the filtering process carried out by OACYCBD and ACYCBD demonstrates its ability to effectively filter noise signals associated with bearing degradation, particularly those with a variable frequency.

In Figure 13b, we introduce a novel health index designed to assess the RUL. Each sample, comprising 20,480 data points, was transformed into sample-level data points using both a health index and RMS. The novel health index excels at measuring bearing degradation in the time domain, capturing distinct health condition stages in different situations, including the normal and failure stages. An initial bearing degradation trend becomes evident at sample number 800 when using OACYCBD and a health index, while ACYCBD+RMS and RMS indicate this trend began at sample number 880. This observation highlights the proposed method’s enhanced sensitivity to bearing degradation when compared with ACYCBD and RMS methods.

Last, in Figure 13c, we address the inverse prediction problem related to memory using the HINN. By framing it as a conditional memory task, we used the complete dataset of 980 samples for both training and testing, as previously described. Results from sample numbers 750 to 980 are presented for degradation predictions. These results demonstrate superior forecasting performance compared with SVM, CNN, and LSTM methods. To quantitatively evaluate performance, we calculated the RMSE, with values of 0.259 for the SVM, 0.317 for the CNN, 0.317 for LSTM, and 0.233 for the HINN. These values highlight HINN’s superior forecasting performance compared with the SVM, CNN, and LSTM methods.

In Figure 14a, the effectiveness of the filtering process carried out by OACYCBD and ACYCBD is evident. The optimal OACYCBD method proved its ability to filter our noise signals associated with bearing degradation, particularly those characterized by variable frequency characteristics.

Continuing with Figure 14b, we utilize the novel HI designed for condition assessment. Each sample, comprising 20,480 data points, is transformed into a single point using both HI and RMS. The novel HI method excels at measuring bearing degradation in the time domain, effectively capturing distinct health condition stages in different scenarios, including the normal stage and the failure stage. Notably, an initial bearing degradation trend is observable at sample number 700 when employing OACYCBD and HI, whereas ACYCBD+RMS and RMS indicate this trend starting after sample number 730. This demonstrates the proposed method’s heightened sensitivity to bearing degradation compared to ACYCBD and RMS methods.

Finally, in Figure 14c, we delve into the inverse prediction problem employing HINN. As previously outlined, we use the complete dataset of 980 samples for both training and testing. Training commenced with data from the first sample, which comprises 20,480 data points, with testing progressing through subsequent samples sequentially. In this instance, data from sample numbers 650 to 980 are presented for degradation predictions. The outcomes unmistakably exhibit superior forecasting performance when compared to SVMs, CNNs, and LSTMs. To provide a quantitative assessment of performance, we compute the RMSE, yielding values of 0.075 for SVMs, 0.049 for CNNs, 0.060 for LSTM, and 0.043 for HINN. These RMSE values underscore HINN’s superior forecasting performance in contrast to SVM, CNN, and LSTM methods.

We applied the proposed methods, namely OACYCBD+HI, ACYCBD+RMS, and RMS, to demonstrate the comparative analysis of different degradation trends. In this section, we explore distinct approaches encompassing filtering methods and health indices to investigate bearing-condition monitoring and predict trends related to RUL of bearings undergoing gradual deterioration and performance loss over time. The results convincingly demonstrate that the proposed method exhibits heightened sensitivity to bearing degradation compared with other methods. Additionally, a HINN was employed to forecast bearing degradation, and the results underscore its superior forecasting performance when contrasted with SVM, CNN, and LSTM methods.

## 6. Conclusions

In this paper, we introduced an OACYCBD method aimed at extracting essential features from mixed vibration signals. These signals were subsequently processed using a novel health index, enabling the analysis of comprehensive health conditions linked to bearing degradation. Additionally, we developed a HINN to predict the health condition of bearing degradation. This holistic approach combines filtering techniques, health index analysis, and predictive modeling to significantly enhance the diagnosis and monitoring of rotating machinery health.

Our experiments have clearly demonstrated the superiority of the proposed method in comparison with ACYCBD, RMS, and LSTM. Several key findings support this conclusion. First, ACYCBD, while capable of identifying fault features with cyclic frequency in vibrating bearing signals, falls short of addressing real-world bearing degradation, which involves the accumulation of fault features at variable cyclic frequencies under conditions of high pressures and speed. OACYCBD addresses this limitation effectively by using a probability density function (PDF) of Monte Carlo to assess condition characteristics and measure subtle differences in vibration signals. Furthermore, it replaces the traditional iteration process of ACYCBD with cross-entropy, resulting in optimized filter coefficients. As a result, OACYCBD significantly outperformed ACYCBD in feature extraction for bearing degradation, providing superior noise signal filtration for variable frequency characteristics.

Second, and following noise signal filtration, we introduced a novel health index that uses peak properties and the square of the arithmetic mean to analyze critical components of equipment degradation and measure various health condition stages. This method is more sensitive to bearing degradation and health condition stages than the ACYCBD+RMS method is.

Third, in our hybrid invertible neural network, we combined INN architecture with LSTM. This model effectively assessed the damage-accumulation process associated with bearing degradation and delivered superior performance in PHM for bearing degradation. In data 1, the results demonstrate superior forecasting performance when compared with SVM, CNN, and LSTM methods. The root mean square error (RMSE) evaluates the performance of different methods for forecasting. The RMSE was 0.799 for SVM, 0.593 for CNN, 0.53 for LSTM, and 0.485 for HINN. In data 2, To quantitatively evaluate performance, we calculated the RMSE, with values of 0.259 for the SVM, 0.317 for the CNN, 0.317 for LSTM, and 0.233 for the HINN. In data 3, the outcomes unmistakably exhibit superior forecasting performance when compared to SVMs, CNNs, and LSTMs. To provide a quantitative assessment of performance, we compute the RMSE, yielding values of 0.075 for SVMs, 0.049 for CNNs, 0.060 for LSTM, and 0.043 for HINN. Notably, it outperformed SVMs, CNNs, and LSTMs in predicting health conditions.

In summary, our research presents a comprehensive hybrid approach that excels in fault diagnosis and health monitoring for rotating machinery. The proposed methods, including advanced filtering, a novel health index analysis, and forecasting models, collectively represent a significant improvement in the recognition of faults in rotating machinery, particularly in scenarios in which bearing degradation involves variable cyclic frequencies.

In the future, multiple sensor inputs can be used to predict the RUL of the bearing. Furthermore, 2D vibration images can be utilized in the future for the prognosis of rotating machinery. Furthermore, the filtering process in the proposed method is computationally expensive. Therefore, in the future, the proposed method can be modified to reduce the computational complexity of the prognosis.

## Figures and Tables

**Table 1:** Performance comparison of model prediction.

RMSE	SVM	CNN	LSTM	HINN
Data1	0.799	0.593	0.530	0.485
Data2	0.259	0.317	0.317	0.233
Data3	0.075	0.049	0.06	0.043

## Data Availability

The data are available upon request.
